# The Role of Optical Coherence Tomography Signal Strength in the Diagnosis and Follow-up of Patients with Posterior Capsular Opacification Treated with Nd:YAG Laser Capsulotomy

**DOI:** 10.4274/tjo.galenos.2019.80378

**Published:** 2020-03-05

**Authors:** Mustafa Vatansever, Erdem Dinç, Özer Dursun, Ufuk Adıgüzel, Ayça Yılmaz, Gülhan Ö. Temel

**Affiliations:** 1 Toros State Hospital, Clinic of Ophthalmology, Mersin, Turkey; 2 Mersin University Faculty of Medicine, Department of Ophthalmology, Mersin, Turkey; 3 Mersin University Faculty of Medicine, Department of Biostatistics and Medical Informatics, Mersin, Turkey

**Keywords:** Optical coherence tomography, posterior capsular opacification, signal strength, visual acuity

## Abstract

**Objectives:**

To investigate the relationship between optical coherence tomography (OCT) signal strength (SS) and visual acuity in patients with posterior capsule opacification (PCO) and evaluate the effect of PCO on retinal thickness measurements.

**Materials and Methods:**

Forty-one eyes of 35 patients who were diagnosed with PCO were included in the study. Patients with any anterior or posterior segment pathology other than PCO were excluded. After ophthalmologic examination, pupil dilation was induced using 0.5% tropicamide and OCT images were acquired. The assessment was repeated 1 month after Nd:YAG laser capsulotomy and postoperative values were compared with baseline values.

**Results:**

The patients’ mean best corrected visual acuity (BCVA) was 0.28±0.13 preoperatively and 0.78±0.09 postoperatively (p<0.0001). Strong positive correlations were observed between BCVA and SS both pre- and postoperatively (p<0.0001 and p=0.01, respectively). Central retinal thickness (CRT) and SS increased significantly postoperatively (p<0.0001 for both). OCT SS and CRT were strongly correlated preoperatively (p=0.001) but not postoperatively (p=0.46).

**Conclusion:**

OCT SS correlates with visual acuity in patients with PCO, and PCO can affect the accuracy of objective data obtained with OCT.

## Introduction

Optical coherence tomography (OCT), introduced into use in 1990, is a low-coherence inferometry instrument that enables non-contact measurement of posterior segment structures.^1^ OCT is widely used because it is a convenient, non-invasive, and sensitive method, and has an important place in the diagnosis and follow-up of many macular diseases.^1,2^ The first OCT devices used in the clinic were time domain OCT (TD-OCT); these were followed by spectral domain OCT (SD-OCT) instruments, which feature faster data acquisition and provide higher resolution images compared to TD-OCT.^3,4,5^

While signal-to-noise ratio was used to express image quality in early devices, this was replaced by signal strength (SS) in later models. With each scan, the instrument displays the SS to the operator, with higher SS corresponding to better image quality. SS may be affected by operator technique, eye and head movements during scan acquisition, and anterior or posterior segment opacities in the eye.^6^

Posterior capsule opacification (PCO) is one of the main factors that can affect SS. PCO is a clinical condition which develops after cataract surgery and results in reduced visual acuity and contrast sensitivity. The gold standard treatment for PCO is neodymium-doped yttrium aluminum garnet (Nd:YAG) laser application.^7,8,9^ The aim of the present study was to investigate the relationship between OCT SS and visual acuity in patients with PCO and evaluate the effect of Nd:YAG laser treatment on OCT measurements in these patients.

## Materials and Methods

The medical records of patients who had Nd:YAG laser capsulotomy to treat PCO were reviewed retrospectively. Nd:YAG laser capsulotomy was performed at Mersin Toros State Hospital in Mersin, Turkey between July 2016 and July 2017. All procedures involving human subjects were performed according to the tenets of the Declaration of Helsinki. Informed consent forms were signed by the patients before enrollment. Forty-one eyes of 35 patients who presented with complaints of low vision after undergoing uncomplicated cataract surgery and were diagnosed with PCO were included in the study. Patients with any anterior or posterior segment pathology other than PCO were excluded. Patients with postoperative macular edema were also excluded from the study. Best corrected visual acuity (BCVA) was evaluated by Snellen chart. After a complete ophthalmologic examination, mydriasis was induced with 0.5% tropicamide. PCO scoring was performed clinically by two blinded specialists using the biomicroscope. Nd:YAG laser capsulotomy was planned for patients with PCO scores of 3 or over. Cross-sectional images were acquired using the macular program in the OCT instrument (Nidek RS 3000; Nidek Co. Ltd., Aichi, Japan). All patients in the study underwent uncomplicated Nd:YAG laser capsulotomy (1.5-2.5 mJ; central opening of 3-4 mm) performed by the same surgeon. All patients received topical prednisolone acetate 4 times a day for 1 week after the procedure. At postoperative 1 month, BCVA was reassessed and postoperative OCT images were acquired after pupil dilation as before. The same experienced operator conducted all OCT scans, and repeated scans in cases with movement-related artifacts. We statistically analyzed the relationship between pre- and postoperative values and OCT SS, and the effect of laser capsulotomy on OCT measurements.

### Statistical Anaylsis

SPSS version 11.5 software was used for statistical analysis. Each parameter was assessed for normal data distribution using the Shapiro-Wilk test. Pre- and postoperative values were compared using Wilcoxon test. Mean and standard deviation were calculated for each parameter. Spearman’s correlation coefficient was calculated for the relationships between parameters. P values <0.05 were accepted as statistically significant.

## Results

**Figure 1:**
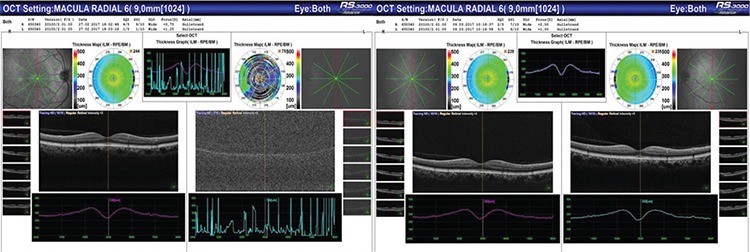
Macular OCT sections before and after Nd:YAG laser treatment of the left eye

**Figure 2:**
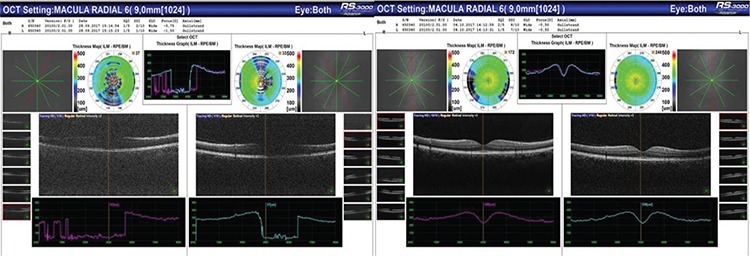
Macular OCT sections before and after Nd:YAG laser treatment of a patient treated in both eyes

The mean age of the study patients (17 women, 18 men) was 62.37±7.2 years. Mean BCVA of the study group was 0.55±0.88 logMAR before Nd:YAG laser surgery and 0.10±1.04 logMAR postoperatively (p<0.0001) (Table 1). Significant positive correlations were observed between BCVA and OCT SS both pre- and postoperatively (p<0.0001 and p=0.01, respectively) (Table 2). Preoperative and postoperative central retinal thickness (CRT) values were 124.49±109.43 and 239.78±57.66 µm (p<0.0001). The mean preoperative SS was 2.98±1.38 and SS increased to 7.29±1.27 postoperatively (p<0.0001) (Table 1). There was also a significant positive correlation between preoperative OCT SS and CRT (p=0.001) (Table 2) (Figure 1 and 2). However, CRT was not correlated with OCT SS after Nd:YAG laser treatment (p=0.46) (Table 2). There was no significant change in intraocular pressure between measurements taken before and after the procedure (p=0.81) (Table 1).

## Discussion

OCT has become an indispensable tool in both the diagnosis and follow-up of macular diseases, glaucoma, and even anterior segment pathologies. However, with the increasing clinical use of OCT instruments, certain technical details involved in data acquisition have gained attention. The most important of these details is the SS obtained during acquisition, which is closely related to the image quality of the scanned sections. Previous studies have shown that OCT measurements can be affected by factors such as age, race, and ocular pathologies, as well as SS.^10,11,12,13,14,15,16,17^

PCO is the most common long-term complication of cataract surgery with intraocular lens implantation, and its prevalence is higher among pediatric patients and patients with trauma, uveitis, and diabetes mellitus.^18,19,20,21^ In a meta-analysis published by Schaumberg et al.,^22^ the incidence of PCO was reported to be 11.8% at postoperative 1 year, 20.7% at 3 years, and 28.4% at 5 years. PCO occurs when epithelial cells remaining in the capsular bag undergo fibrous metaplasia, proliferation, and migration. However, the cause of this process is not fully understood. The postoperative inflammatory response is thought to increase lens epithelial cell proliferation.^23,24^

Hougaard et al.^25^ suggested that PCO obscures retinal details by reducing signal quality, but reported no significant change between macular thickness measurements taken before and after laser treatment. In another study, it was observed that SS and visual acuity were correlated in PCO patients prior to Nd:YAG laser treatment, but PCO did not have an effect on macular measurements.^26^ Considering both of these studies, it should be noted that Hougaard et al.^25^ had a small sample number, while the postoperative SS in the other study was low (6.3). Another study by Kara et al.^27^ reported that retinal nerve fiber layer thickness measurements significantly increased in patients with dense PCO following laser treatment. However, the same study reported no such relationship in cases without dense PCO. Cagini et al.^28^ evaluated the quality and accuracy of measurements taken with both TD-OCT and SD-OCT devices in patients with PCO. The authors stated that PCO lowered the quality of measurements taken using TD-OCT, while the same effect was not observed in SD-OCT. They also observed that measurements taken with TD-OCT were lower than those taken with SD-OCT. Taken collectively, these studies lead to the conclusion that measurements can be affected in patients with dense PCO. In the present study, measurements were taken using SD-OCT in patients with PCO score of 3 or greater. Our results showed that despite taking measurements with SD-OCT, the presence of dense PCO could affect the values obtained, and that signal power was correlated with visual acuity and measured values. This finding demonstrates that the accuracy of measured values is affected as PCO increases in severity.

OCT SS was 6 or less before the Nd:YAG laser treatment and increased after laser treatment in all patients. These findings indicate that OCT SS can provide objective data when determining the Nd:YAG laser indications in patients with PCO. The increase in OCT SS after Nd:YAG laser capsulotomy may provide an objective measure of whether laser treatment was completed successfully.

## Conclusion

In conclusion, OCT SS can provide information about opacity density in addition to that gained through biomicroscopic evaluation. It can also provide more objective data compared to subjective examination findings and help prevent unnecessary procedures. Considering that OCT is a non-invasive, easy, and rapid imaging technique, adding OCT SS as a parameter in the evaluation of patients with PCO may be beneficial.

## Figures and Tables

**Table 1:**
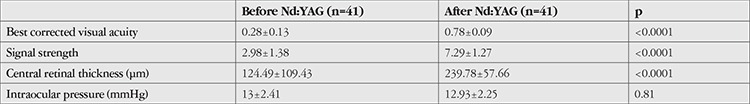
Best corrected visual acuity, signal strength, central retinal thickness, and intraocular pressure values before and after Nd:YAG laser treatment

**Table 2:**
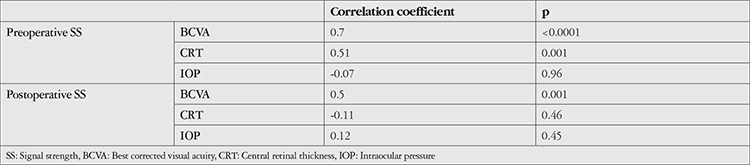
Comparison of best corrected visual acuity, signal strength, central retinal thickness, and intraocular pressure values before and after Nd:YAG laser treatment
